# Study on the Permeability and Absorption Performance of the Crotch Layer in Seamless Knitted Period Underwear

**DOI:** 10.3390/ma17051119

**Published:** 2024-02-29

**Authors:** Wenqi Chen, Zimin Jin, Si Chen, Chengxiao Fang, Cong Zheng

**Affiliations:** 1 College of Textile Science and Engineering, Zhejiang Sci-Tech University, Hangzhou 310018, China; cwq3302@163.com (W.C.); icecilechen@163.com (S.C.); 2 Zhejiang Bangjie Holding Group Co., Ltd., Yiwu 322009, China; fangcx@bangjie.cn; 3 Zhejiang Xinlan Textile Co., Ltd., Lanxi 321100, China; hellangelsky@sina.com

**Keywords:** period underwear, seamless knitting, liquid strike-through time, penetration amount, rewet, liquid absorption capacity

## Abstract

During the physiological period, women have the problem of lateral and posterior leakage, and they expect to have period underwear that can reduce lateral and posterior leakage. This study is combined with menstrual needs, and in the crotch penetration layer, three types of yarns are used, seaweed viscose yarn, apocynum viscose yarn, and viscose yarn, as well as two fabric structures: honeycomb-shaped convex–concave stitching and grid-shaped convex point stitching. In the crotch absorption layer, three types of yarns are used, modal yarn, bamboo yarn, and viscose yarn, as well as two fabric structures: plush stitching and plain stitching. The above two parts establish a sample scheme according to full-factor experimental tests, and 12 knitted fabric samples were knitted. The experimental data were analyzed through SPSS one-way ANOVA. The results indicate that in terms of veil raw materials, the crotch penetration layer with seaweed viscose yarn has better penetration performance, while the crotch absorption layer with bamboo yarn has better absorption performance. In terms of fabric structure, the crotch penetration layer with grid-shaped convex point stitching has better penetration performance, while the crotch absorption layer with plush stitching has better absorption performance. This study provides a theoretical basis for the development of period underwear.

## 1. Introduction

During the menstrual period, period underwear can provide necessary protection for women. Period underwear can effectively absorb and prevent menstrual blood leakage, maintain women’s hygiene and cleanliness, and reduce the chance of bacterial growth, thereby reducing risk of gynecological diseases for women. At the same time, it can reduce the pollution of bed sheets, clothing, and other items by women during the menstrual period, making it convenient for cleaning and management. Period underwear can also alleviate women’s discomfort during the menstrual period and can be reused, allowing women to spend their menstrual period more comfortably [1,2].

At present, physiological underwear mainly adopts a layered and partitioned design, using different partitions to absorb and penetrate. Julie Sygiel started researching menstrual underwear in 2008 and founded the Dead Kate Company in 2012. She invented a three-layer structure of physiological underwear, with two layers absorbing liquid and one layer preventing liquid from leaking onto clothes. The raw material for the suction layer is polyester microfiber, and the raw material for the leakproof layer is nylon and Lycra. There is also a layer of liquid mosquito repellent on the physiological underwear, which can hold three teaspoons of liquid at once. Thinx Company was founded in 2011, and Sygiel and Agrawal designed underwear with layers. The first layer (body layer) of the underwear is made of materials that prevent blood backflow, the second layer is made of antibacterial materials, the third layer is made of blood-absorbing materials, and the fourth layer is made of leakproof materials [3]. Currently, many researchers have researched the comfort, penetration, absorption performance, and warmth retention of women’s underwear [4,5,6,7,8], but there is relatively little research on the design and development of physiological underwear from the perspective of yarn and fabric organization.

With the continuous improvement in women’s understanding of physiological health, their requirements for the quality of sanitary products are also increasing. A neat appearance and comfortable hand feel are just general requirements. They also hope that the design of period underwear conforms to ergonomics and has strong permeability and high absorption [9,10,11]. The crotch layer is a key part of period underwear, which is divided into a crotch penetration layer, a crotch absorption layer, and a crotch bottom layer. The crotch bottom layer is to prevent leakage, which is difficult to achieve through veil selection and structural design. The penetration performance of the crotch penetration layer and the absorption performance of the absorption crotch layer are the keys to the penetration and absorption performance of period underwear, which is crucial for meeting the physiological health needs of women. Therefore, this study investigates the effects of yarn raw materials and fabric structure on the penetration and absorption performance of period underwear crotch pieces.

In this study, seaweed viscose yarn, apocynum viscose yarn, and viscose yarn were selected as materials for the crotch penetration layer, and modal yarn, bamboo yarn, and viscose yarn were selected as materials for the crotch absorption layer. Select honeycomb-shaped convex–concave stitching and grid-shaped convex point stitching served as the fabric structure of the crotch penetration layer, and plush stitching and plain stitching were used as the fabric structure of the crotch absorption layer. In response to the need for women’s period underwear, a 28-needle SM8-TOP2 electronic seamless forming knitting circular machine was used to knit. The liquid strike-through time, penetration amount, rewet, and liquid absorption capacity were tested. The influence of raw materials and fabric structure on the penetration and absorption performance of the crotch fabric layer was studied, providing a theoretical reference for the development of women’s period underwear that reduces side leakage.

## 2. Materials and Methods

### 2.1. Materials

#### 2.1.1. Yarn

This study mainly explores the influence of different yarn and knitted fabric structures on the permeability of the crotch penetration layer and the absorption of the crotch absorption layer. The material of yarn is important in the comfort of seamless knitted women’s physiological underwear [12]. Seaweed viscose yarn has good permeability, flexibility, breathability, flame retardance, and so on, and can improve the permeability of the fabric [13]. The middle of apocynum fiber is hollow, which can enhance the permeability of the fabric. It contains various flavonoids, has good antibacterial properties [14], and is good for human health [15]. Therefore, the crotch penetration layer veil adopts seaweed viscose yarn and apocynum viscose yarn.

The modal yarn has the characteristics of an excellent flexibility, size stability, high strain recovery rate, alkali resistance, good moisture absorption, high strength, and high wet modulus [16], which can improve the absorption performance of the fabric. Bamboo yarn has high fracture strength and good water absorption performance [17,18], which can improve the absorption performance of fabrics. Therefore, the crotch absorption layer veil adopts modal and bamboo yarn.

The lining yarn was made of 2.22 tex/3.33 tex (20D/30D) nylon/spandex-covered yarn provided by Yiwu Huading Nylon Co., Ltd. (Yiwu, China) to improve the elasticity of the designed knitted fabric. The fineness of the veil yarn in the crotch penetration layer and the crotch absorption layer is 14.58 tex (40 s). The specific yarn and specifications of the sample veil are shown in Table 1. Viscose was used as a blank control.

#### 2.1.2. Fabric Structure Design

The fabric structure plays an important role in the wearability of seamless knitted fabrics for women’s period underwear [19,20]. The honeycomb-shaped convex–concave stitch is designed to form a honeycomb-like concave–convex shape, imitating sanitary napkins’ penetration layer pattern. The honeycomb design can enhance the beauty of the fabric, enhance the concave–convex effect, increase the three-dimensional sense of the pattern, and, to a certain extent, increase the surface area of the fabric and enhance its permeability. The grid-shaped convex point stitch can increase the fabric’s breathability and beautify its appearance by designing a grid-like protrusion structure. The grid shape can increase the direct contact area of the human body, thereby enhancing the permeability. Therefore, the fabric structure of the crotch penetration layer adopts the honeycomb-shaped convex–concave stitch and the grid-shaped convex point stitch.

**Figure 1:**
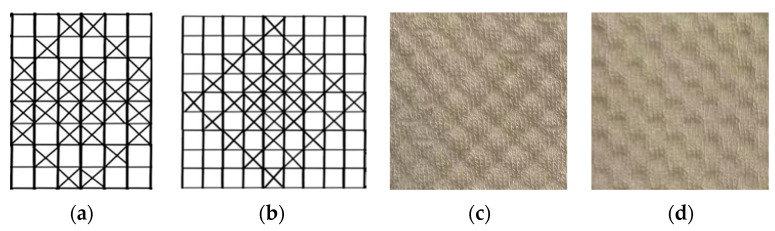
(**a**) Honeycomb-shaped convex–concave stitch; (**b**) grid-shaped convex point stitch; (**c**) knitting samples for honeycomb-shaped convex–concave stitch; (**d**) knitting samples for grid-shaped convex point stitch.

The coil diagram is suitable for simpler fabric structures. The honeycomb-shaped convex–concave stitch and grid-shaped convex point stitch are complex, and the pattern is large, making it unsuitable to use the coil diagram for representation. The knitting diagram is too complex, so the knitting diagram is not used. Therefore, a pattern design diagram is used to represent the honeycomb-shaped convex–concave stitch and the grid-shaped convex point stitch of the front pattern and pattern of the fabric. The honeycomb-shaped convex–concave stitch pattern is shown in Figure 1a, and the grid-shaped convex point stitch pattern is shown in Figure 1b. The knitting samples for honeycomb-shaped convex–concave stitching and grid-shaped convex point stitching are shown in Figure 1c,d.

The plush stitch is a patterned stitch composed of plain stitching and loop coils with elongated settling arcs. It has good insulation and moisture absorption properties and can enhance the fabric’s absorption. The plain stitch fabric is composed of continuous coils of the same unit arranged in one direction. The fabric is thick and full, and has good absorption properties. Therefore, the fabric structure of the crotch absorption layer adopts the plush stitch and the plain stitch.

#### 2.1.3. Establishment of Sample Scheme

To analyze the penetration of the crotch penetration layer sample, two factors were set for the fabric of the crotch penetration layer: Factor A is different types of veil yarns, divided into three levels, namely seaweed viscose, apocynum viscose, and viscose. Factor B is the knitted fabric structure, divided into two levels, namely honeycomb-shaped convex–concave stitching and grid-shaped convex point stitching. The full-factor test design method was adopted [21] and the specific fabric sample scheme is shown in Table 2.

To analyze the absorption of the crotch absorption layer sample, two factors were set for the crotch absorption layer fabric: Factor A is different types of veil yarns, divided into three levels, namely modal, bamboo, and viscose. Factor B is the knitted fabric structure, divided into two levels: plush stitching and plain stitching. The full-factor test design method was adopted, and the specific fabric sample scheme is shown in Table 3.

The knitting machine used to complete sample knitting is SM8-TOP2S of Santoni, the gauge is E28, the feeder count is 16, and the diameter of the cylinder is 14 inches.

### 2.2. Methods

#### 2.2.1. Liquid Strike-Through Time

Experimental instrument: YG814D liquid penetration tester.

Experimental procedure: As there is no testing standard for the liquid penetration time of fabrics, the test is carried out by the standard GB/T 24218.13-2010 Textiles-Test methods for nonwovens Part 13: Repeated liquid strike-through time [22].

#### 2.2.2. Penetration Amount

Experimental instrument: FFZ194 penetration performance tester.

Experimental procedure: As there is no testing standard for the penetration amount of fabrics, the test is carried out by standard GB/T 30133-2013 The specification of coverstock for sanitary absorbent pads [23].

Experimental calculation results:

Calculate the penetration amount of each sample according to Formula (1).

m = m_1_ − m_0_ (1)

among them, m_0_ is the mass of the filter paper before the experiment, and m_1_ is the mass of the filter paper after the experiment. m is the penetration amount of the sample, expressed as the mass of the penetration test solution, in grams (g).

#### 2.2.3. Rewet

Experimental instrument: Test-specific standard pressure block.

Experimental procedure: As there is no testing standard for the rewet of fabrics, the test is carried out by standard GB/T 30133-2013 The specification of coverstock for sanitary absorbent pads.

Experimental calculation results:

The rewet of each sample is calculated according to Formula (2).

G = G_2_ − G_1_ (2)

among them, G_1_ represents the quality of the filter paper before the experiment, and G_2_ represents the quality of the filter paper after the experiment. G is the rewet of the sample, expressed in grams (g) as the mass of liquid that has permeated the sample and returned to the filter paper.

#### 2.2.4. Liquid Absorption Capacity

Experimental instrument: LBX-116 non-woven fabric water absorption performance tester.

Experimental procedure: As there is no testing standard for the liquid absorption capacity of fabrics, the test is carried out by the standard GB/T 24218.6-2010 Textiles-Test methods for nonwovens-Part 6: Absorption [24].

Experimental calculation results:

The liquid absorption capacity (LAC) of each sample is calculated according to Formula (3).

(3) $$LAC=\frac{m_{n}-m_{k}}{m_{k}}\times 100\%$$

among them, m_k_ is the mass of the sample after humidity adjustment, in grams (g). m_n_ is the mass of the test after aspiration, in grams (g). LAC represents the ability of the sample to absorb liquid, which is a percentage.

## 3. Results

### 3.1. Study on Fabric Structure Parameters and Permeability of Seamless Knitted Fabric with Crotch Penetration Layer

#### 3.1.1. Study on Fabric Structure Parameters and Liquid Strike-Through Time of Seamless Knitted Fabric with Crotch Penetration Layer

According to the standard GB/T 24218.13-2010 Textiles-Test methods for nonwovens Part 13: Repeated liquid strike-through time, liquid strike-through time tests were conducted on samples #1–#6. The results of the liquid strike-through time tests for each seamless knitted fabric are shown in Table 4.

The liquid strike-through time is an important indicator for measuring the permeability of fabrics. The liquid quickly penetrates from the surface of the crotch penetration layer to the crotch absorption layer. The shorter the liquid strike-through time, the faster the liquid penetration speed of the crotch penetration layer, and the better the crotch penetration performance [25]. According to the data analysis in Table 4, the larger the liquid contact surface area, the shorter the liquid penetration time of the fabric, and the better the penetration performance of the fabric. The raw material for the veil is seaweed viscose yarn, and sample #7 with honeycomb-shaped convex–concave stitching has the shortest liquid strike-through time and good permeability. The raw material for the veil is viscose yarn, and sample #12 with grid-shaped convex point stitching has the longest liquid penetration time and poor permeability. The order of liquid penetration time from short to long is #1 < #2 < #4 < #5 < #3 < #6.

Conduct one-way ANOVA using SPSS software to further explore the relationship between yarn raw materials, fabric structure, and liquid strike-through time. Due to the presence of two independent variables, veil raw material and fabric structure, and six liquid strike-through time data samples in this experiment, there is a problem with the small sample size of experimental data, which may affect the analysis of experimental results. Therefore, during the analysis of variance, 10 sets of experimental data were entered into the database, totaling 60 data samples. The analysis results are shown in Table 5.

From the data analysis in Table 5, the significance level of the material is *p* < 0.05, the significance level of the fabric structure is *p* < 0.05, and the significance level of the material*structure is *p* < 0.05. This indicates that the single factor of the veil raw material and fabric structure has a significant impact on the liquid strike-through time, and the interaction between the veil raw material and the fabric structure is significant. Secondly, the data of the three follow a normal distribution and have statistical significance.

**Figure 2:**
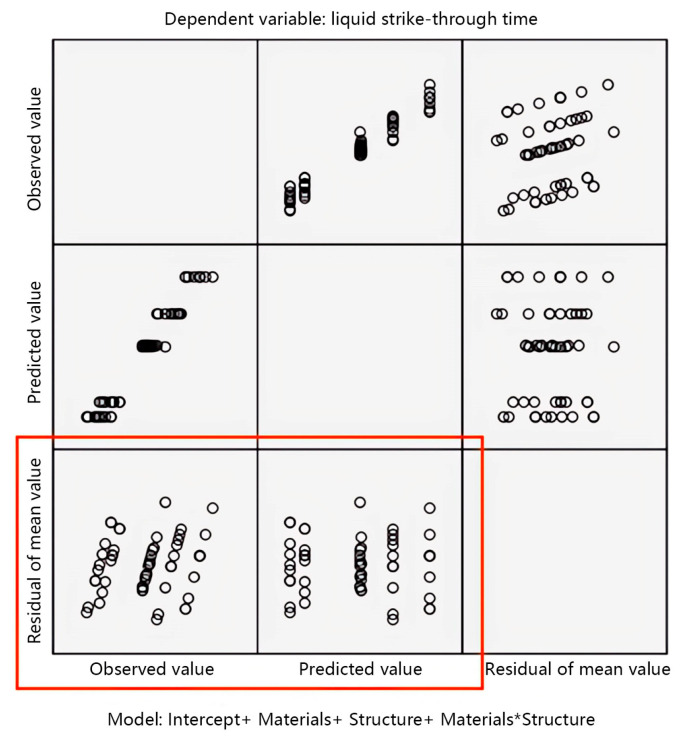
Standard residual plot of liquid strike-through time.

This study uses the normality condition to test the accuracy of the variance analysis results of fabric structural parameters and liquid strike-through time. The test results are shown in Figure 2.

The accuracy of the observation test results is mainly achieved by observing the scatter plot as the vertical axis in the standard residual plot. From the standard residual plot in Figure 2, it can be observed that the distribution of each point in the scatter plot of the residual is uniform and there is no obvious pattern to follow, indicating that the data approximately follow or follow a normal distribution with homogeneous variance. The analysis of variance method can continue to be used.

**Figure 3:**
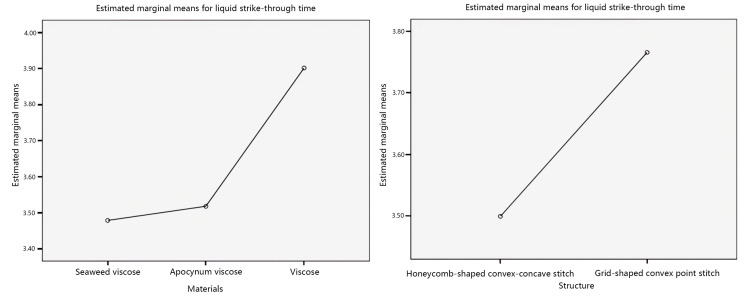
Main effects’ plot of liquid strike-through time.

Further, investigate the influence of individual factors on liquid strike-through time, and the main effect plots of the estimated marginal mean of liquid strike-through time are shown in Figure 3.

Analyzing the main effect plots in Figure 3, with the veil material as the independent variable, the estimated marginal average of the liquid strike-through time of seaweed viscose yarn is relatively low. The estimated marginal average of the liquid strike-through time of honeycomb-shaped convex–concave stitching is relatively low, with the fabric structure as the independent variable. Secondly, with the veil material as the independent variable, the order of liquid strike-through time from short to long is seaweed viscose yarn < apocynum viscose yarn < viscose yarn. The order of liquid strike-through time from short to long, with fabric structure as the independent variable, is honeycomb-shaped convex–concave stitching < grid-shaped convex point stitching.

**Figure 4:**
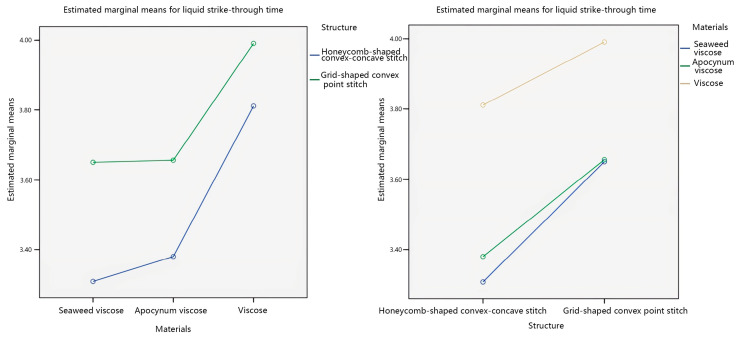
Interaction plots of liquid strike-through time.

This study considers the interaction between the veil material and the fabric structure, which affects the liquid strike-through time of seamless knitted fabrics. Therefore, a pairwise comparison was made between the veil material and the fabric structure, as shown in Figure 4.

Analyzing Figure 4, it can be observed from the plots that in the interaction plots of the two factors with the veil material as the independent variable, there is no intersection between the honeycomb-shaped convex–concave stitch line and the grid-shaped convex point stitch line. In the interaction plots of the two factors with fabric structure as the independent variable, there is an intersection between seaweed viscose yarn creases and apocynum viscose creases. This indicates that there is an interaction between the veil material and the fabric structure regarding the liquid strike-through time, and the interaction is significant. In addition, the fabric structure has a more significant effect on the liquid strike-through time of seamless knitted fabrics than the veil material, which is consistent with the significance analysis results of the material*structure obtained from Table 5.

#### 3.1.2. Study on Fabric Structure Parameters and Penetration Amount and Rewet of Seamless Knitted Fabric with Crotch Penetration Layer

According to standard GB/T 30133-2013 The specification of coverstock for sanitary absorbent pads, samples #1–#6 were tested for the penetration amount and rewet, and the penetration performance test results of each seamless knitted fabric are shown in Table 6.

The penetration amount and rewet are two important indicators for measuring the permeability of fabrics. The more the penetration amount, the less rewet, indicating better permeability of the fabric. According to the data analysis in Table 6, the raw material for the veil is seaweed viscose yarn, and the penetration amount of #4 with grid-shaped convex point stitching is the highest. The raw material for the veil is viscose yarn, and the penetration amount of #6 with grid-shaped convex point stitching is the lowest. The raw material for the veil is seaweed viscose yarn, with grid-shaped convex point stitching having the least amount of rewet. The raw material for the veil is viscose yarn, with honeycomb-shaped convex–concave stitching having the most amount of rewet. The order of the seamless knitted fabric penetration amount from more to less is #4 > #5 > #1 > #2 > #3 > #6, and the order of seamless knitted fabric rewet from less to more is #4 < #2 < #6 < #1 < #5 < #3.

1. Fabric structure parameters and penetration amount analysis:

Conduct one-way ANOVA using SPSS software to further explore the relationship between yarn raw materials, fabric structure, and penetration amount. Due to the presence of two independent variables, veil raw material and fabric structure, and six penetration amount data samples in this experiment, there is a problem with a small sample size of experimental data, which may affect the analysis of experimental results. Therefore, during the analysis of variance, five sets of experimental data were entered into the database, totaling 30 data samples. The analysis results are shown in Table 7.

From the data analysis in Table 7, the significance level of the veil raw material is *p* < 0.05, the significance level of the fabric structure is *p* < 0.05, and the significance level of the material*structure is *p* < 0.05, indicating that the single factor of the veil raw material and fabric structure has a significant impact on the penetration amount. The interaction between the veil raw material and the fabric structure is significant. Secondly, the three data categories follow a normal distribution and have statistical significance.

**Figure 5:**
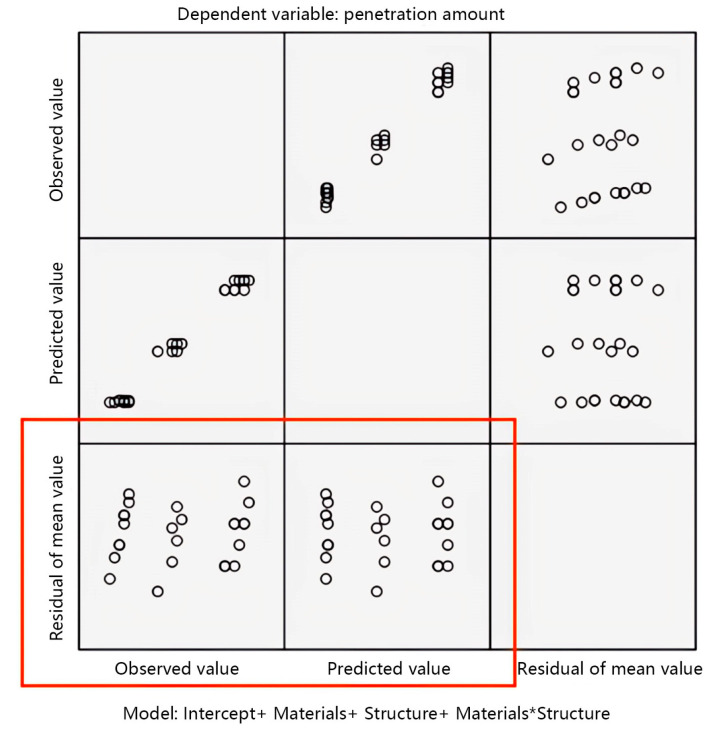
Standard residual plot of penetration amount.

This study uses the normality condition to test the accuracy of the variance analysis results of fabric structural parameters and penetration amount. The test results are shown in Figure 5.

The accuracy of the observation test results is mainly achieved by observing the scatter plot as the vertical axis in the standard residual plot. From the scatter plot of the mean residual in Figure 5, it can be observed that the distribution of each point in the scatter plot of the residual is uniform and there is no obvious pattern to follow, indicating that the data approximately follow or follow a normal distribution with homogeneous variance. The analysis of variance method can continue to be used.

**Figure 6:**
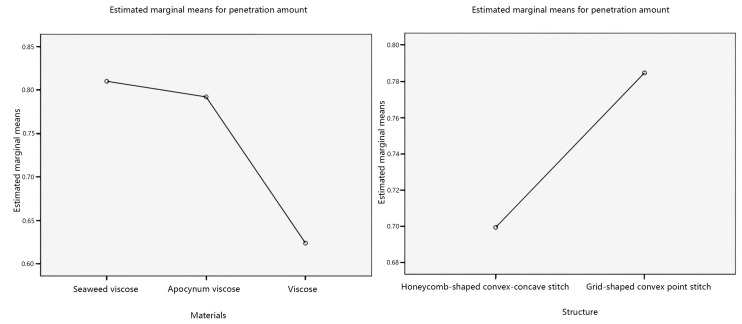
Main effects’ plot of penetration amount.

Further, investigate the influence of individual factors on the penetration amount, and the main effect plots of the estimated marginal mean values of the penetration amount are shown in Figure 6.

Analyzing the main effect diagram in Figure 6, with the veil material as the independent variable, the estimated marginal mean value of the penetration amount of seaweed viscose yarn is relatively high. The estimated marginal mean value of the penetration amount of the grid-shaped convex point stitch is relatively high, with the fabric structure as the independent variable. Secondly, with the veil raw material as the independent variable, the order of the penetration amount from more to less is seaweed viscose yarn > apocynum viscose yarn > viscose yarn. The order of the penetration amount from more to less, with the fabric structure as the independent variable, is grid-shaped convex point stitching > honeycomb-shaped convex–concave stitching.

**Figure 7:**
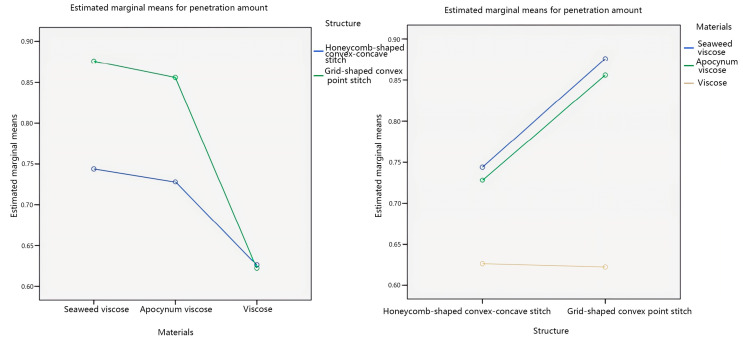
Interaction plots of penetration amount.

This study considers the interaction between the veil raw material and the fabric structure, which affects the penetration amount of seamless knitted fabrics. Therefore, a pairwise comparison was made between the veil raw material and the fabric structure, as shown in Figure 7.

Analyzing Figure 7, it can be observed from the plots that in the interaction plots of the two factors with the veil material as the independent variable, there is an intersection between the honeycomb-shaped convex–concave stitch line and the grid-shaped convex point stitch line. In the two-factor interaction diagram with fabric structure as the independent variable, there is no intersection between the three broken lines of seaweed viscose yarn, apocynum viscose yarn, and viscose yarn. This indicates that there is an interactive effect between the veil raw material and the fabric structure on the penetration amount, and the interaction is significant. In addition, the influence of the veil raw material on the penetration amount of seamless knitted fabrics is more significant than that of the fabric structure, which is consistent with the significance analysis results of the material*structure obtained from Table 7.

2. Fabric structure parameters and rewet analysis:

Conduct one-way ANOVA using SPSS software to further explore the relationship between veil raw material, fabric structure, and rewet. Due to the presence of two independent variables, veil raw material and fabric structure, and six samples of rewet data in this experiment, there is a problem of an insufficient sample size of experimental data, which may affect the analysis of experimental results. Therefore, during the analysis of variance, five sets of experimental data were entered into the database, totaling 30 data samples. The analysis results are shown in Table 8.

From the data analysis in Table 8, it can be concluded that the significance level of veil raw materials is *p* < 0.05, the significance level of fabric structure is *p* < 0.05, and the significance level of materials*structure is *p* < 0.05. This indicates that the single factor of veil raw materials and fabric structure has a significant impact on the amount of rewet, and the interaction between veil raw materials and fabric structure is significant. Secondly, the data of the three categories follow a normal distribution and have statistical significance.

**Figure 8:**
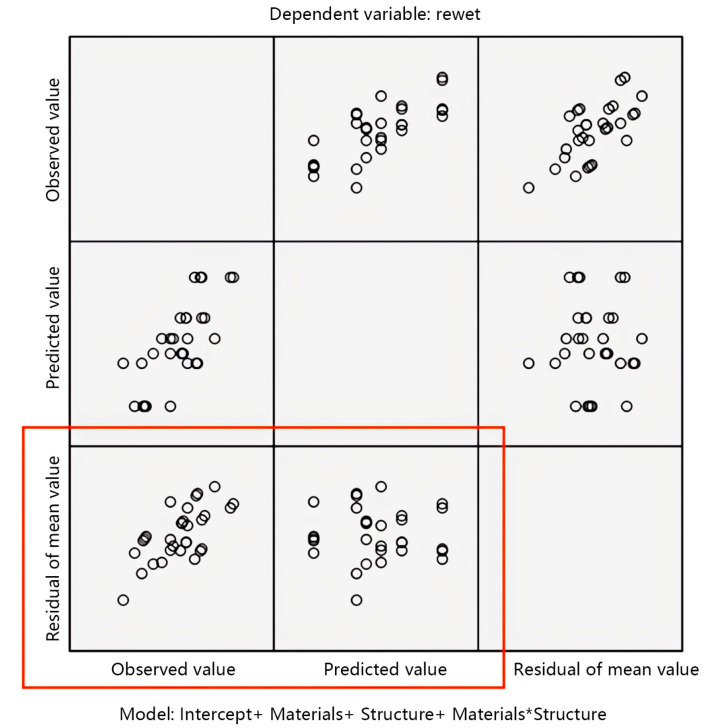
Standard residual plot of rewet.

This study uses the normality condition to test the accuracy of the variance analysis results of fabric structural parameters and rewet. The test results are shown in Figure 8.

The accuracy of the observation test results is mainly achieved by observing the scatter plot as the vertical axis in the standard residual plot. From the scatter plot of the mean residual in Figure 8, it can be observed that the distribution of each point in the scatter plot of the residual is uniform and there is no obvious pattern to follow, indicating that the data approximately follow or follow a normal distribution with homogeneous variance. The analysis of variance method can continue to be used.

**Figure 9:**
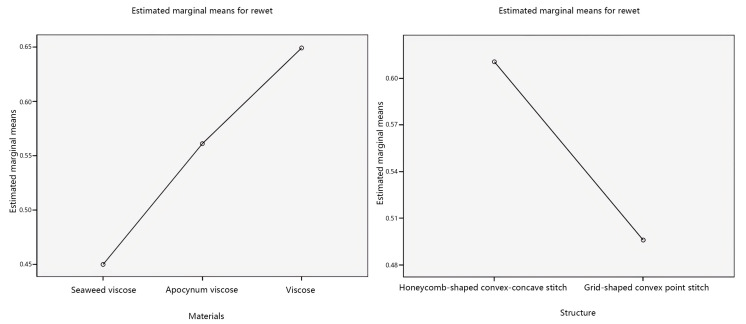
Main effects’ plot of rewet.

The impact of individual factors on the rewet was further explored, and the main effect plots of the estimated marginal mean of rewet were created, as shown in Figure 9.

Analyzing the main effect diagram in Figure 9, with the veil raw material as the independent variable, the estimated marginal average of the rewet of seaweed viscose yarn is relatively low. The estimated marginal average of the rewet of the grid-shaped convex point stitch is relatively low, with the fabric structure as the independent variable. Secondly, with the veil raw material as the independent variable, the order of rewet from more to less is seaweed viscose yarn < apocynum viscose yarn < viscose yarn. The order of rewet from more to less, with fabric structure as the independent variable, is grid-shaped convex point stitching < honeycomb-shaped convex–concave stitching.

**Figure 10:**
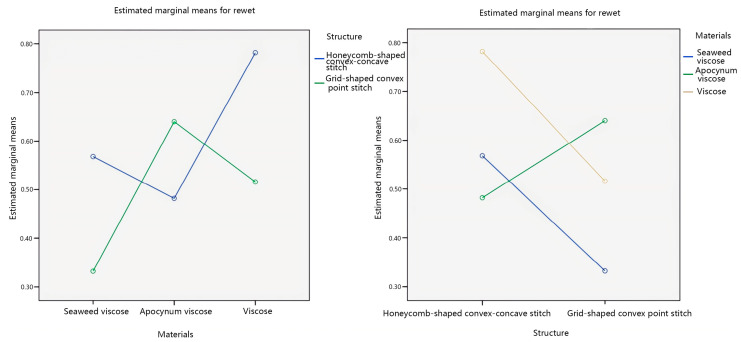
Interaction plots of rewet.

This study considers the interaction between the veil material and the fabric structure, which affects the rewet of seamless knitted fabrics. Therefore, a pairwise comparison was made between the veil material and the fabric structure, as shown in Figure 10.

Analyzing Figure 10, it can be observed from the plots that in the interaction plots of the two factors with the veil material as the independent variable, there is an intersection between the honeycomb-shaped convex–concave stitch line and the grid-shaped convex point stitch line. In the interaction plots of the two factors with fabric structure as the independent variable, there are intersections between apocynum viscose creases and three creases: seaweed viscose yarn and viscose yarn. The interaction between veil raw materials and fabric structure regarding the rewet of seamless knitted fabrics is significant, which means that there is a significant interaction between the two significant factors of veil raw materials and fabric structure, which affects the results of rewet of seamless knitted fabrics. This is consistent with the significance analysis results of veil raw materials and fabric structure obtained from Table 8.

### 3.2. Study on Fabric Structure Parameters and Liquid Absorption Capacity of Seamless Knitted Fabric with Crotch Absorption Layer

According to the standard GB/T 24218.6-2010 Textiles-Test methods for nonwovens-Part 6: Absorption, liquid absorption capacities were tested on samples #7–#12. The results of the liquid absorption capacity for each seamless knitted fabric are shown in Table 9.

The liquid absorption capacity is an important indicator for measuring the absorption performance of fabrics. The larger the liquid absorption capacity, the better the liquid absorption performance of the fabric. According to the data analysis in Table 9, the liquid absorption capacity of #8 with bamboo yarn as the raw material for the veil and looped structure is the highest. The veil raw material is modal yarn, and the liquid absorption capacity of #10 with a flat needle structure is the smallest. The order of liquid absorption capacity from most to least is #8 > #7 > #9 > #12 > #11 > #10.

Further explore the relationship between yarn raw materials, fabric structure, and liquid absorption capacity through a variance analysis using SPSS software. Due to the existence of two independent variables, veil raw material and fabric structure, and six samples of liquid absorption capacity data in this experiment, there is a problem of a too small sample size of experimental data, which may affect the analysis of experimental results. Therefore, during the analysis of variance, five sets of experimental data were entered into the database, totaling 30 data samples. The analysis results are shown in Table 10.

From the data analysis in Table 10, the significance level of the veil raw material is *p* < 0.05, the significance level of the fabric structure is *p* < 0.05, and the significance level of the material*structure is *p* < 0.05, indicating that the single factor of the veil raw material and fabric structure has a significant impact on the liquid absorption capacity, and the interaction between the veil raw material and the fabric structure is significant. Secondly, the data of the three categories follow a normal distribution and have statistical significance.

**Figure 11:**
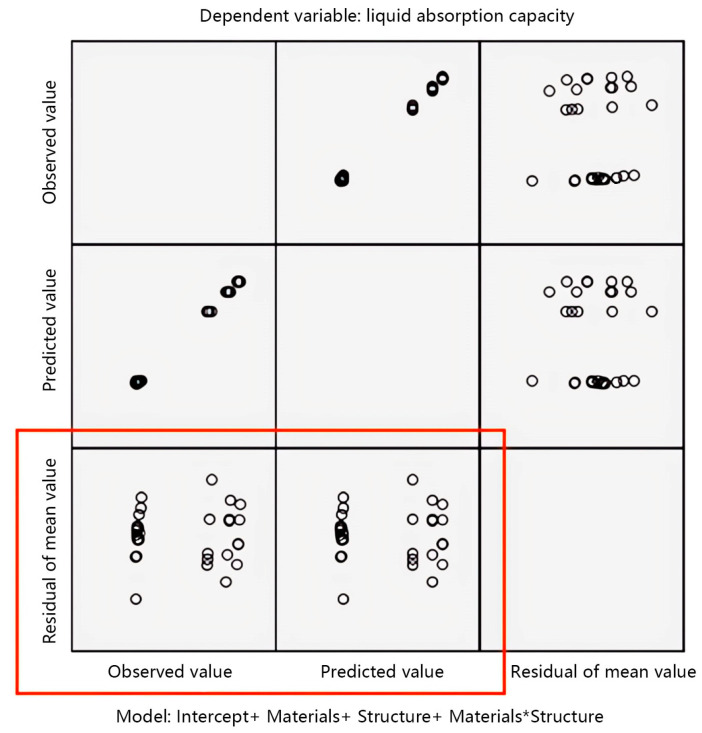
Standard residual plot of liquid absorption capacity.

This study uses the normality condition to test the accuracy of the variance analysis results of fabric structural parameters and liquid absorption capacity. The test results are shown in Figure 11.

The accuracy of the observation test results is mainly achieved by observing the scatter plot as the vertical axis in the standard residual plot. From the scatter plot of the mean residual in Figure 11, it can be observed that the distribution of each point in the scatter plot of the residual is uniform and there is no obvious pattern to follow, indicating that the data approximately follow or follow a normal distribution with homogeneous variance. The analysis of variance method can continue to be used.

**Figure 12:**
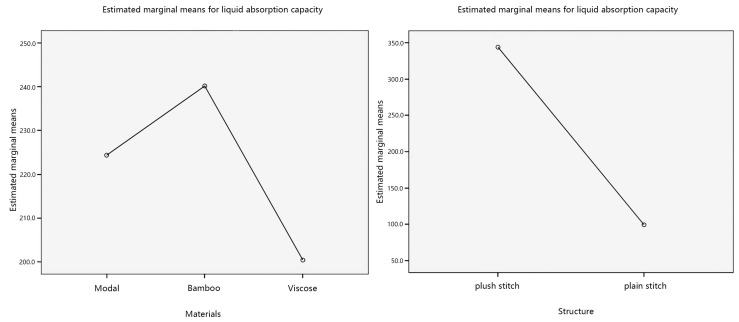
Main effects’ plot of liquid absorption capacity.

Further, investigate the influence of individual factors on the liquid absorption capacity, and the main effect plots of the estimated marginal mean of liquid absorption capacity are shown in Figure 12.

Analyzing the main effect diagram in Figure 12, with the veil material as the independent variable, the estimated marginal average value of liquid absorption capacity of bamboo yarn is relatively high. The estimated marginal mean value of liquid absorption capacity of plush stitching is relatively high, with fabric weave as the independent variable. Secondly, with the veil raw material as the independent variable, the order of liquid absorption capacity from more to less is bamboo yarn > modal yarn > viscose yarn. The order of liquid absorption capacity from more to less, with fabric structure as the independent variable, is plush stitching > plain stitching.

**Figure 13:**
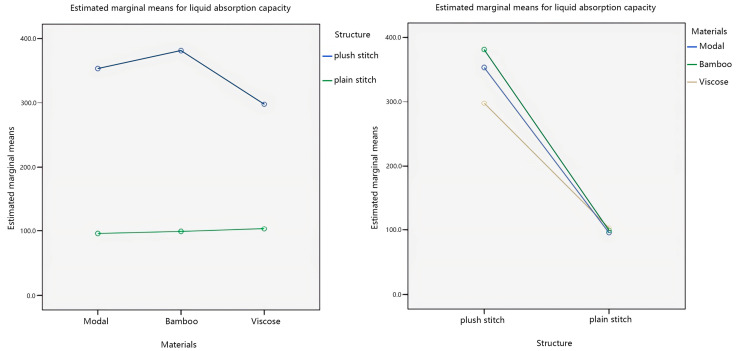
Interaction plots of liquid absorption capacity.

This study considers the interaction between the veil raw material and the fabric structure, which affects liquid absorption capacity of seamless knitted fabrics. Therefore, a pairwise comparison was made between the veil raw material and the fabric structure, and the results are shown in Figure 13.

Analyzing Figure 13, it can be observed from the plots that in the interaction plots of the two factors with the veil raw material as the independent variable, there is no intersection between the two-fold lines of plush stitching and plain stitching. In the interaction plots of the two factors with fabric structure as the independent variable, there is an intersection between the three lines of modal yarn, bamboo yarn, and viscose yarn. This indicates that there is an interaction between the liquid absorption capacity of the veil material and the fabric structure, and the interaction is significant. In addition, the influence of the fabric structure on liquid absorption capacity of seamless knitted fabrics is more significant than that of the veil raw material, which is consistent with the significance analysis results of the material*structure obtained from Table 10.

## 4. Discussion and Conclusions

This study conducted penetration tests on the crotch penetration layer, and absorption tests on the crotch absorption layer. SPSS software was used to conduct a variance analysis on the relationship between the veil raw material, fabric structure, and properties of seamless knitted fabrics. The conclusions are as follows:

For the crotch penetration layer, the shorter the liquid strike-through time, the faster liquid diffuses from the crotch penetration layer to the crotch absorption layer, and the better the fabric penetration performance. According to the results of liquid strike-through time, it can be seen that the single factor of veil raw material and fabric structure has a significant impact on liquid strike-through time, and the interaction between veil raw material and fabric structure is significant. In terms of veil raw materials, the order of liquid strike-through time from short to long is seaweed viscose yarn < apocynum viscose yarn < viscose yarn. In terms of fabric structure, the order of liquid strike-through time from short to long is honeycomb-shaped convex–concave stitching < grid-shaped convex point stitching.

For the crotch penetration layer, the more the penetration amount and less rewet, the better fabric penetration performance. The main effect of a single factor on the amount of penetration is significant, and the interaction between the veil raw material and fabric structure is significant. In terms of veil raw materials, the order of the penetration amount from more to less is seaweed viscose yarn > apocynum viscose yarn > viscose yarn. In terms of fabric structure, the order of the penetration amount from more to less is grid-shaped convex point stitching > honeycomb-shaped convex–concave stitching. According to the results of the penetration amount data, it can be seen that the main effect of a single factor on the penetration amount is significantly affected by the veil raw material and fabric structure, and the interaction between the veil raw material and fabric structure is significant. In terms of veil raw materials, the order of rewet from less to more is seaweed viscose yarn < apocynum viscose yarn < viscose yarn. In terms of fabric structure, the order of rewet from less to more is grid-shaped convex point stitching < honeycomb-shaped convex–concave stitching.

For the crotch absorption layer, the more the liquid absorption capacity, the better fabric absorption performance. The single factor of veil raw materials and fabric organization has a significant impact on the liquid absorption capacity, and the interaction between veil raw materials and fabric structure is significant. In terms of veil raw materials, the order of liquid absorption capacity from more to less is bamboo yarn > modal yarn > viscose yarn. In terms of fabric structure, the order of liquid absorption capacity from more to less is plush stitching > plain stitching.

The absorption and penetration properties of seamless knitted fabrics are related to the moisture absorption performance of fabrics. There are a large number of hydrophilic groups such as carboxyl and hydroxyl groups in the molecular structure of seaweed viscose fibers, which can absorb water in the air. In addition, there are a large number of amorphous regions distributed, so they have excellent moisture absorption. Seaweed viscose seamless knitted fabrics have good absorption and permeability performance. The chemical composition of bamboo fibers is mainly cellulose, belonging to the cellulose type I crystalline structure with small molecular weight. A single bamboo fiber has a circular cross-section and a small inner cavity, so its moisture absorption performance is better. Grid-shaped convex point stitching and plush stitching have a larger contact area with the human body, which gives them better absorption and permeability performance.

In this study, we focus on the influence of yarn raw material and fabric structure on the penetration and absorption performance of seamless knitted period underwear. This study combines the physiological needs of women. It aims to develop the crotch absorption layer with excellent absorption performance and the crotch penetration layer with penetration performance, laying a theoretical foundation for developing dry, comfortable, and anti-side-leakage period underwear.

## Figures and Tables

**Table 1:** Veil materials and specifications.

Part	Veil Type	Supplier
crotch penetration layer	seaweed viscose	Kangkang Textile Technology Co., Ltd. (Yantai, China)
crotch penetration layer	apocynum viscose	Baicao New Materials Co., Ltd. (Qingdao, China)
crotch penetration layer	viscose	Huarui Textile Co., Ltd. (Puyang, China)
crotch absorption layer	modal	Huarui Textile Co., Ltd. (Puyang, China)
crotch absorption layer	bamboo	Huarui Textile Co., Ltd. (Puyang, China)
crotch absorption layer	viscose	Huarui Textile Co., Ltd. (Puyang, China)

**Table 2:** Crotch penetration layer fabric specimen scheme.

Sample Number	Yarn Raw Materials (A)	Fabric Structures (B)
#1	14.58 tex (40 s) seaweed viscose	honeycomb-shaped convex–concave stitch
#2	14.58 tex (40 s) apocynum viscose	honeycomb-shaped convex–concave stitch
#3	14.58 tex (40 s) viscose	honeycomb-shaped convex–concave stitch
#4	14.58 tex (40 s) seaweed viscose	grid-shaped convex point stitch
#5	14.58 tex (40 s) apocynum viscose	grid-shaped convex point stitch
#6	14.58 tex (40 s) viscose	grid-shaped convex point stitch

**Table 3:** Crotch absorption layer fabric specimen scheme.

Sample Number	Yarn Raw Materials (A)	Fabric Structures (B)
#7	14.58 tex (40 s) modal	plush stitch
#8	14.58 tex (40 s) bamboo	plush stitch
#9	14.58 tex (40 s) viscose	plush stitch
#10	14.58 tex (40 s) modal	plain stitch
#11	14.58 tex (40 s) bamboo	plain stitch
#12	14.58 tex (40 s) viscose	plain stitch

**Table 4:** Test results of liquid strike-through time for samples #1–#6.

Sample Number	Liquid Strike-Through Time (5 mL/s)
#1	3.31
#2	3.48
#3	3.81
#4	3.65
#5	3.74
#6	3.99

**Table 5:** Variance analysis of liquid strike-through time.

Source	Class III Sum of Squares	Freedom	Mean Square	F	Significance
Modified model	3.303 ^a^	5	0.661	226.089	0.000
Intercept	791.776	1	791.776	270,984.316	0.000
Materials	2.175	2	1.088	372.246	0.000
Structure	1.061	1	1.061	363.242	0.000
Materials*Structure	0.066	2	0.033	11.356	0.000
Error	0.158	54	0.003		
Total	795.237	60			
Corrected total	3.461	59			

^a^ R square = 0.954 (after adjustment, R square = 0.950).

**Table 6:** Test results of penetration amount and rewet for samples #1–#6.

Sample Number	Penetration Amount (g)	Rewet (g)
#1	0.74	0.57
#2	0.73	0.48
#3	0.63	0.78
#4	0.88	0.33
#5	0.86	0.64
#6	0.62	0.52

**Table 7:** Variance analysis of penetration amount.

Source	Class III Sum of Squares	Freedom	Mean Square	F	Significance
Modified model	0.295 ^a^	5	0.059	318.962	0.000
Intercept	16.517	1	16.517	89,280.649	0.000
Materials	0.210	2	0.105	568.865	0.000
Structure	0.055	1	0.055	295.207	0.000
Materials*Structure	0.030	2	0.015	80.937	0.000
Error	0.004	24	0.000		
Total	16.816	30			
Corrected total	0.299	29			

^a^ R square = 0.985 (after adjustment, R square = 0.982).

**Table 8:** Variance analysis of rewet.

Source	Class III Sum of Squares	Freedom	Mean Square	F	Significance
Modified model	0.577 ^a^	5	0.115	5.894	0.001
Intercept	9.185	1	9.185	468.799	0.000
Materials	0.199	2	0.099	5.075	0.015
Structure	0.099	1	0.099	5.033	0.034
Materials*Structure	0.280	2	0.140	7.143	0.004
Error	0.470	24	0.020		
Total	10.233	30			
Corrected total	1.048	29			

^a^ R square = 0.551 (after adjustment, R square = 0.458).

**Table 9:** Test results of liquid absorption capacity for samples #7–#12.

Sample Number	Liquid Absorption Capacity (%)
#7	353
#8	381.4
#9	297.7
#10	95.7
#11	98.9
#12	103.1

**Table 10:** Variance analysis of liquid absorption capacity.

Source	Class III Sum of Squares	Freedom	Mean Square	F	Significance
Modified model	467,583.722 ^a^	5	93,516.744	4835.072	0.000
Intercept	1,473,773.016	1	1,473,773.016	76,198.109	0.000
Materials	8003.069	2	4002.534	206.890	0.000
Structure	449,330.408	1	449,330.408	23,231.615	0.000
Materials*Structure	10,250.245	2	5125.122	264.983	0.000
Error	464.192	24	19.341		
Total	1,941,820.930	30			
Corrected total	468,047.914	29			

^a^ R square = 0.999 (after adjustment, R square = 0.999).

## Data Availability

Data are contained within the article.
